# Terpenoid Mixtures as Repellents Against the American Cockroach: Their Synergy and Low Toxicity Against Non-Target Species

**DOI:** 10.3390/insects17010065

**Published:** 2026-01-05

**Authors:** Hataichanok Passara, Tanapoom Moungthipmalai, Chamroon Laosinwattana, Sirawut Sittichok, Kouhei Murata, Mayura Soonwera

**Affiliations:** 1 Office of Administrative Interdisciplinary Program on Agricultural Technology, School of Agricultural Technology, King Mongkut’s Institute of Technology Ladkrabang, Ladkrabang, Bangkok 10520, Thailand; hataichanok.pa@kmitl.ac.th (H.P.); 64604012@kmitl.ac.th (T.M.); chamroon.la@kmitl.ac.th (C.L.); 2 School of Agriculture and Cooperatives, Sukhothai Thammathirat Open University, Nonthaburi 11120, Thailand; sirawut.sit@stou.ac.th; 3 School of Agriculture, Tokai University, Kumamoto 862-8652, Japan; kmurata@agri.u-tokai.ac.jp

**Keywords:** *Periplaneta americana*, synergistic repellent effect, terpenoid binary mixture, geranial + *trans*-cinnamaldehyde, non-target earthworm and guppies

## Abstract

The repellent activities (*Periplaneta americana*) of lone and binary mixtures of terpenoids—geranial, *trans*-anethole, and *trans*-cinnamaldehyde—against adult American cockroaches were evaluated in this study. The respective efficacies of these mixtures were compared with that of 12% (*w*/*w*) DEET. Safety bioassays for all formulations on non-target species, namely, earthworms (*Eudrilus eugeniae*) and guppy fish (*Poecilia reticulata*), were conducted to identify natural compounds with repellent efficacy equal to or surpassing that of DEET while ensuring ecological safety for non-target organisms such as fish and earthworms. All mixtures (RC_50_ of 0.3 to 1.6 µL/cm^3^) were more effective than all lone terpenoids (RC_50_ of 6.2 to 9.1 µL/cm^3^) and DEET (RC_50_ of 3.0 µL/cm^3^), demonstrating strong synergy, with an increased repellent value (IV) of 79 to 96%. The strongest repellency, 98.0% at 72 h and an RC_50_ of 0.3 µL/cm^3^, was achieved using geranial + *trans*-cinnamaldehyde (1:1). This mixture was 5.9 to 10 times more effective as a repellent than DEET. The toxicity of every lone terpenoid and terpenoid mixture to non-target species was low; hence, these mixtures can be considered safe, whereas DEET was highly toxic to non-target species (100% mortality). The terpenoid mixture geranial + *trans*-cinnamaldehyde showed strong repellency against *P. americana.*

## Simple Summary

The American cockroach (*Periplaneta americana* (L.)) is a serious vector of human diseases and allergens, especially in children. Safe and effective natural insecticides and repellents are needed to safeguard human health and environmental safety, especially in sensitive areas where American cockroaches reside, such as the sewers and drains of hospitals and nursing homes, residential homes, and cafeterias or other food-handling places. Natural repellents, namely, terpenoids from plant essential oils, are understood to be strong candidates for cockroach management. In this study, the repellent efficacy of lone terpenoids and terpenoid mixtures—geranial, *trans*-anethole, and *trans*-cinnamaldehyde—as repellents against adult cockroaches was investigated and compared with those of DEET (*N*,*N*-diethyl-meta-toluamide) and distilled water. The mixture of geranial + *trans*-cinnamaldehyde (1:1) was found to be the most effective cockroach repellent and was more effective than all lone terpenoids and DEET, with a long repellent time and low repellent concentration. All binary mixtures showed a high increase in repellency. All treatments except DEET were safe for the two non-target species considered: earthworms (*Eudrilus eugeniae*) and guppies (*Poecilia reticulata*). Our findings demonstrate the potential of a binary mixture of geranial + *trans*-cinnamaldehyde (1:1) to be developed into valuable natural repellents for American cockroach control.

## 1. Introduction

**Figure 1:**
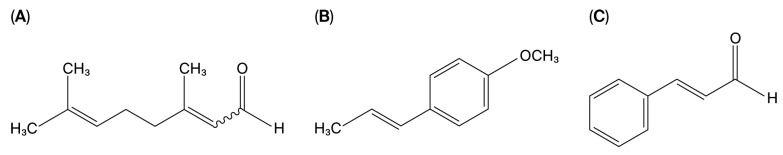
Chemical structures (Sigma-Aldrich): geranial (**A**), *trans*-anethole (**B**), and *trans*-cinnamaldehyde (**C**).

American cockroaches (*Periplaneta americana* (L.)) have a global distribution and are a significant urban pest, particularly in tropical and subtropical climates. They are very common in homes, cafeterias, dining establishments, kitchens, and other food preparation spaces, and restrooms [1,2]. More significantly, these cockroaches endanger human health by mechanically spreading at least 56 species of human-pathogenic bacteria, fungi, protozoa, and parasitic worms that cause food poisoning, diarrhea, dysentery, and other kinds of infection [3,4]. Some people, particularly small children, pregnant women, and nursing mothers, are allergic to the antigens in their excrement, which can lead to eczema, asthma, and allergic rhinitis [4,5]. Additionally, these cockroaches represent a serious cause of annoyance, causing worry and psychological suffering [4,5]. Cockroach populations are extremely challenging to manage effectively. The most common way to control them was, until recently, the use of synthetic insecticides and repellents, e.g., DEET, which has been used against German cockroach (*Blattella germanica*) and American cockroach [6,7,8,9,10]. DEET (*N*,*N*-diethyl-m-toluamide) has long been the most common synthetic repellent used to control insect vectors, particularly cockroaches [10,11]. Unfortunately, prolonged use of DEET has led to a number of issues, including risks to human health, persistent environmental pollution, and toxicity to non-target creatures [10,11,12,13,14]. Using fewer synthetic repellents and insecticides is a recognized strategy in the management of cockroaches [15,16]. One approach to address this problem is to develop innovative natural repellents based on bioactive compounds from plant essential oils (EOs), especially terpenoids—geranial, *trans*-anethole, and *trans*-cinnamaldehyde (the chemical structures are shown in Figure 1)—as alternatives to synthetic repellents [15,16,17]. They are characterized by strong multifunctional properties, strong repellency synergism, and multiple modes of action [17,18]. These natural repellents can be considered safe substitutes for synthetic repellents and insecticides in sensitive areas such as homes, nursing homes, and nurseries, posing no threat to young children, patients, pregnant women, or nursing mothers [19,20]. Terpenoid-based repellents are extremely safe to both humans and other non-target creatures, environmentally benign, and effective while tending to evaporate quickly in the environment [20,21,22].

Terpenoids are major bioactive compounds of EOs. They can be divided into several groups, including acyclic monoterpenoids (linalool, geranial, and geraniol) and phenylpropanoids (cinnamaldehyde, eugenol, anethole, and myristicin) [23]. Geranial, an acyclic monoterpenoid abundantly present in the essential oils of lemon myrtle (*Backhousia citriodora*), mountain pepper (*Litsea citrate*), and lemongrass (*Cymbopogon citratus*) [17,18], has demonstrated insecticidal activity against the eggs, larvae, and pupae of the dengue mosquito (*Aedes aegypti*) [17,18,22]; the adult housefly (*Musca domestica*) [24,25]; and the larvae of the cabbage looper (*Trichoplusia ni*) [26]. *Trans*-anethole is a phenylpropanoid found in the EOs of various plants, most notably star anise (*Illicium verum*), fennel (*Foeniculum vulgare*), and anise (*Pimpinella anisum*) [23]. It has shown repellent and insecticidal activities against houseflies, the *Ae. aegypti* mosquito, and the rusty grain beetle (*Cryptolestes ferrugineus*) [25,26,27]. *Trans*-cinnamaldehyde, a phenylpropanoid and an aromatic aldehyde, is a major constituent of EOs from *Cinnamomum* spp. [28]. It acts as a repellent against adult American cockroaches and adult *Ae. aegypti* [28,29] and was demonstrated to have strong larvicidal and adulticidal activities against mosquito vectors (*Ae. albopictus* and *Ae. aegypti*) [29,30]. Furthermore, several researchers have reported that mixtures of an EO and a terpenoid exhibit higher repellency efficacy against the American cockroach and other vector insects than lone-compound formulations. Binary mixtures (1:1) of the EOs of star anise (*I. verum*) + cinnamon (*C. verum*) and geranial + *trans*-anethole have been shown to have a strong repellent effect (100%) against adult American cockroaches—1.9–2.0 times more repellent than lone-compound formulations [19,28]. In addition, most binary terpenoid mixtures have been found to be non-toxic to tested non-target species (namely, the guppy, *Poecilia reticulata*; molly, *P. latipinna*; dwarf honeybee, *Apis florea*; honeybee, *Apis mellifera*; stingless bee, *Tetragonula pagdeni*; and earthworm, *Eudrilus eugeniae*) [19,31]. Cockroach repellents, even if applied in sewers or drains, can leach into soil or water systems. Earthworms represent soil organisms, while guppies represent aquatic organisms. Testing ensures that repellents will not harm beneficial species if residues spread beyond cockroach habitats. However, the emphasis of the above-cited studies was on mixtures of an EO and an EO constituent. Consequently, the aim of this study was to assess the repellency of lone terpene formulations and binary mixtures of terpenoids—geranial, *trans*-anethole, and *trans*-cinnamaldehyde—against the adult American cockroach (*P. americana*). These three terpenoids were selected because they have been widely reported to possess therapeutic, food-preserving, insecticidal, and repellent properties and to be non-toxic to humans, the environment, and non-target species [19,31,32,33,34]. The synergistic repellent effects and biosafety of binary mixtures against the African nightcrawler earthworm (*E. eugeniae*) and an aquatic predator, the guppy (*P. reticulata*), were evaluated. Guppy fish are a biological pest control agent for mosquito larvae and serve as a model organism for scientific research in ecology, evolution, and behavior due to their rapid breeding and adaptation [35]. Earthworms are known as “ecosystem engineers and farmer’s friends” because they improve soil structure and increase decomposition and soil nutrients. Earthworm populations also serve as an ecological index for assessing soil quality [36]. The African nightcrawler earthworm is widespread in the agroecosystem throughout Asia, including Thailand. Both non-target species were indicator species commonly used in ecotoxicology [35,36,37]. The findings from this study provide useful data for the further development of terpenoid-based repellents for preventing the American cockroach.

## 2. Materials and Methods

### 2.1. American Cockroach Rearing

American cockroaches were collected from Ban Yut Sup market in Ladkrabang, Thailand (13.7200° N, 100.7400° E), and reared in glass jars (225 mm in diameter by 350 mm) at 25 ± 2 °C, 75 ± 3% relative humidity, 0–10 optimal lux, and a 12:12 (light–dark) photoperiod at the School of Agricultural Technology, King Mongkut’s Institute of Technology Ladkrabang, Bangkok, Thailand (13.8436° N, 100.5077° E). They were fed dog food combined with milk powder and 10% glucose syrup, as well as 5% multivitamin syrup soaked in cotton wool. After 6 months of rearing, they grew through the stages of their life cycle (incomplete metamorphosis)—i.e., from the oothecal stage to the nymphal and adult stages. Only one-month-old adults were used in the repellent bioassay [28]. In the experiment, a total of 2000 healthy, adult, morphologically intact cockroaches of comparable size and weight were selected from the containing jar. Five replicates were conducted, with ten individuals per replicate.

### 2.2. Chemicals and Treatments

The 98% geranial (Cas-No: 5392-40-5, the main ingredient of *Cymbopogon citratus* DC. Stapf EO), *trans*-anethole (Cas-No: 4180-23-8, the main ingredient of *Illicium verum* Hook. f EO), and *trans*-cinnamaldehyde (Cas-No: 14371-10-9, the main ingredient of *Cinnamomum verum* J. Presl. EO) were purchased from Sigma-Aldrich Ltd. (Saint Louis, MO, USA). Their chemical structures are shown in Figure 1, and the chemical compositions of the EOs from *Cymbopogon citratus*, *Cinnamomum verum*, and *Illicium verum* are shown in Table 1. The 1% stock solutions were prepared by adjusting the volume of the solvent (70% (*v*/*v*) ethyl alcohol), which was supplied by Siribuncha Company Limited, located in Phra Khanong, Bangkok, Thailand. The stock solutions were stored in sealed brown bottles. The tested lone terpene formulations were geranial, *trans*-anethole, and *trans*-cinnamaldehyde at 0.45, 0.9, 1.8, 3.6, and 7.2 µL/cm^3^. The tested binary terpenoid mixtures were geranial+ *trans*-anethole (1:1), geranial + *trans*-cinnamaldehyde (1:1), and *trans*-anethole + *trans*-cinnamaldehyde (1:1) at 0.45, 0.9, 1.8, 3.6, and 7.2 µL/cm^3^. At these concentrations, the lone terpenoid formulations were shown to have repellent effects against adult houseflies [19,31]. The positive control, 12% *w*/*w* DEET, was prepared by dilution with 70% (*v*/*v*) ethyl alcohol (which was supplied by CP Consumer Products Company Limited, located in Minburi, Bangkok, Thailand) at 0.45, 0.9, 1.8, 3.6, and 7.2 µL/cm^3^. The negative control, distilled water, was acquired from the School of Food Industry at King Mongkut’s Institute of Technology, Ladkrabang (KMITL), Bangkok, Thailand.

### 2.3. Bioanalysis of Repellent Activity

**Figure 2:**
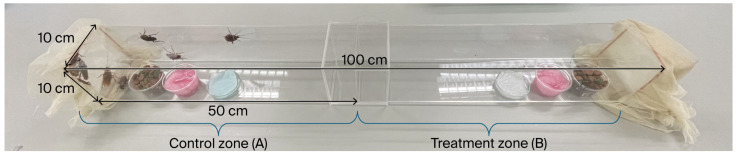
A plastic box with a control zone (**A**) and a treatment zone (**B**) for American cockroach repellency testing.

The cockroach repellency assay was evaluated by using the double-choice method (Figure 2). The test box was designed especially for repellency bioassay and conducted using an open-sided, two-chamber plastic box (100 cm × 10 cm × 10 cm). The box was divided into two equal chambers: the first chamber contained a test formulation (terpenoids + ethyl alcohol), but the second chamber contained only ethyl alcohol, with no tested formulation. In the repellency bioassay, a sheet of cotton wool was placed on a small glass plate (diameter: 5 cm; height: 1.5 cm) in each chamber (Figure 2). In the treatment zone, cotton wool was impregnated with the test formulations at concentrations of 0.45, 0.9, 1.8, 3.6, and 7.2 µL/cm^3^, while 0.45, 0.9, 1.8, 3.6, and 7.2 µL/cm^3^ of ethyl alcohol was dropped onto the cotton wool and placed in the control zone. DEET and distilled water were used concurrently with the tested formulations. To prevent starvation, identical food and water sources were provided in both the control and treatment zones. For each replicate, ten adult cockroaches were introduced into the chamber through the treatment zone. The number of cockroaches present in the control zone and treatment zone was recorded at 1, 6, 12, 24, 48, and 72 h. Five replicates were performed for each treatment. Repellency rates were calculated according to the methods described by Passara et al. [15] and Sittichok et al. [16].

Repellency rate (%RR) = [A − B/A + B] × 100(1)

where A is the total number of American cockroach adults in the control zone, and B is the total number of American cockroach adults in the treatment zone.

Repellency rates were determined at 1, 6, 12, 24, 48, and 72 h. The repellency index (RI) was determined with the following formula:

RI = %RR_treat_/%RR_Deet_, (2)

where %RR_treat_ is the %RR of the tested formulations, and %RR_Deet_ is the % repellency of DEET.

An RI of less than 1 means that the treatment is less potent than DEET, while an RI of more than 1 means that the treatment is more potent than DEET.

The increased repellent value (IV) was calculated according to the formula reported by [19,31].

(3) $$\%\mathrm{IV}=\frac{\left[{\mathrm{RC}}_{50}\;\mathrm{single}\;\mathrm{terpenoid}\;1\;+{\mathrm{RC}}_{50}\;\mathrm{single}\;\mathrm{terpenoid}\;2]\;-\;{\mathrm{RC}}_{50}\;\mathrm{binary}\;\mathrm{mixtures}\right]}{\left[{\mathrm{RC}}_{50}\;\mathrm{single}\;\mathrm{terpenoid}\;1\;+{\mathrm{RC}}_{50}\;\mathrm{single}\;\mathrm{terpenoid}\;2\;+{\mathrm{RC}}_{50}\;\mathrm{binary}\;\mathrm{mixtures}\right]}\times 100,$$

where RC_50 single_ is the 50% repellency concentration of the lone terpenoid at 72 h, and RC_50 binary_ is the 50% repellency concentration of the binary mixture at 72 h.

A relative synergy is indicated by IV: if IV is more than 50%, a synergistic impact is present; if IV is less than 50%, no synergy is present [19,25,30,31]. 

### 2.4. Toxicity Bioassay for Non-Target Species

#### 2.4.1. Guppie Bioassay

Guppies were bought from a guppy fish farm in Thailand—specifically, an organic farm in Prachuap Khiri Khan’s Province (11.5290° N, 99.6369° E). In line with Soonwera et al. [19] and Passara et al. [31], we assessed each formulation’s toxicity to guppies. A total of 2000 fish were reared for this study, and 1200 fish were selected and kept in a 500 × 500 × 300 mm plastic container with 85 L of clean water at 25 ± 4 °C, with 71 ± 2.8% relative humidity, 12-h light and 12-h dark intervals, pH 6.5–7.0, dissolved oxygen ≥ 5 mg/L, and 75–100 mg/L water hardness. This bioassay involved both male and female guppies. A plastic container containing ten mature guppies (diameter: 300 mm; height: 200 mm) was filled with five liters of water. Three concentrations of each treatment—0.1, 0.5, and 1.0 mL/L [31]—were tested. Five replicates (ten fish/one replication) of each treatment concentration were conducted concurrently with the negative (distilled water) and positive controls (DEET). For 15 days after treatment, guppy mortality and aberrant behaviors resulting in fish death were noted.

#### 2.4.2. Earthworm Bioassay

The OECD guideline [38] and Passara et al. [31] were all adhered to in the protocol for the toxicity assay against earthworms (*E. eugeniae*). A total of 2000 earthworms were collected from the KMITL organic farm on 25 May 2025, and 1200 earthworms were separated into a black plastic container that measured 850 mm in diameter and 250 mm in height. Three concentrations of each treatment—0.1, 0.5, and 1.0 mL/kg [31]—were tested. The container held 5 kg of test soil, which included organic fertilizer, cow manure, coconut husk, and organic soil in a 1:1:1:1 ratio at a pH of 6.5–7.0, with 65% soil moisture [31]. The container was maintained at 25 ± 2 °C with 12 h light and 12 h dark intervals. After 1 kg of test soil had been mixed with 1 mL of each treatment, the test soil was placed in a black plastic container that measured 250 mm in diameter and 200 mm in height. Ten earthworms were then added. Biological response consistency was used as an assessment approach. Earthworm responses were observed, and the absence of differences in behavior or response across spatial positions indicated that the substance was not locally concentrated. Five replicates (ten earthworms/one replication) of each treatment were conducted concurrently with the negative (distilled water) and positive controls (DEET). At 1, 5, 10, and 15 days after therapy, the mortality rates were noted. 

#### 2.4.3. Mortality Rate (%M) and Biosafety Ratio (BR) Calculation for Guppy Fish and Earthworm

The following formula was used to calculate the mortality rate (%M):

Mortality rate (%M) = D/T × 100,(4)

where D is the number of dead guppies or earthworms, and T is the number of treated guppies or earthworms.

The following formula was used to determine the Biosafety Ratio (BR) for the guppies and earthworms [19,31]:

(5) $$\mathrm{BR}\;=\;\frac{{\mathrm{LT}}_{50\mathrm{test}}}{{\mathrm{LT}}_{50\mathrm{DEET}}},$$

where LT_50 test_ is the 50% lethal time of the tested formulation, and LT_50 DEET_ is the 50% lethal time of DEET.

A BR of less than 1 indicates that the EO treatment is relatively hazardous to the non-target species, whereas a BR of more than 1 indicates that the treatment is largely benign.

### 2.5. Statistical Analysis

IBM’s SPSS Statistical Software Package version 28 (Armonk, NY, USA) was used for statistical analysis. The bioassays were of a completely randomized design (CRD). Tukey’s test (*p* < 0.01) was used to assess the mean differences across different treatment groups, and one-way analysis of variance (ANOVA) was performed to investigate the mean repellency rate of all treatments and the mean mortality rate of the non-target bioassay [39]. Probit analysis was used to assess the concentration of any particular treatment that repelled 25, 50, and 90% (RC_25_, RC_50_, and RC_90_) of cockroaches after 72 h of exposure. Simple regression was employed to assess the efficacy of the repellent against adult American cockroaches, using generalized linear models with a binomial distribution. R^2^, a correlation coefficient, was used to assess linearity [40].

## 3. Results

### Repellent Activity

**Figure 3:**
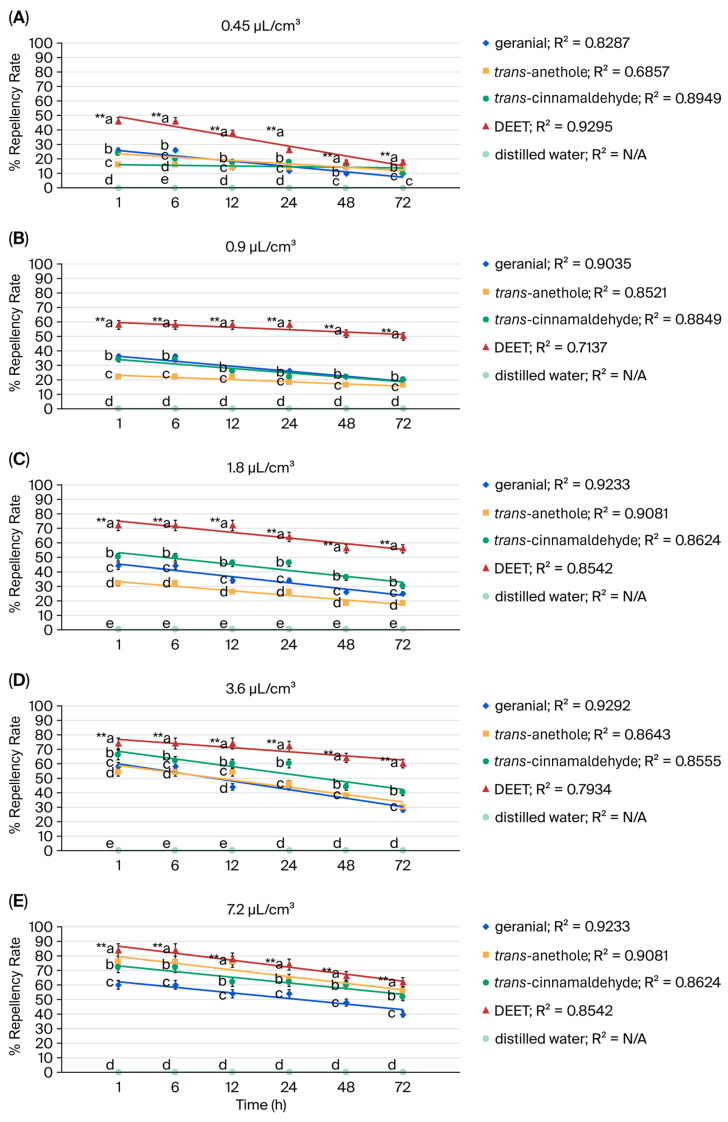
Repellency rate versus exposure time for single terpenoids and DEET against the American cockroach (*P. americana*) at 0.45 µL/cm^3^ (**A**), 0.9 µL/cm^3^ (**B**), 1.8 µL/cm^3^ (**C**), 3.6 µL/cm^3^ (**D**), 7.2 µL/cm^3^ (**E**). Values that are accompanied by different letters (a–e) show significant differences between the treatments. ** for *p* < 0.01, the coefficient of determination value (R^2^).

**Figure 4:**
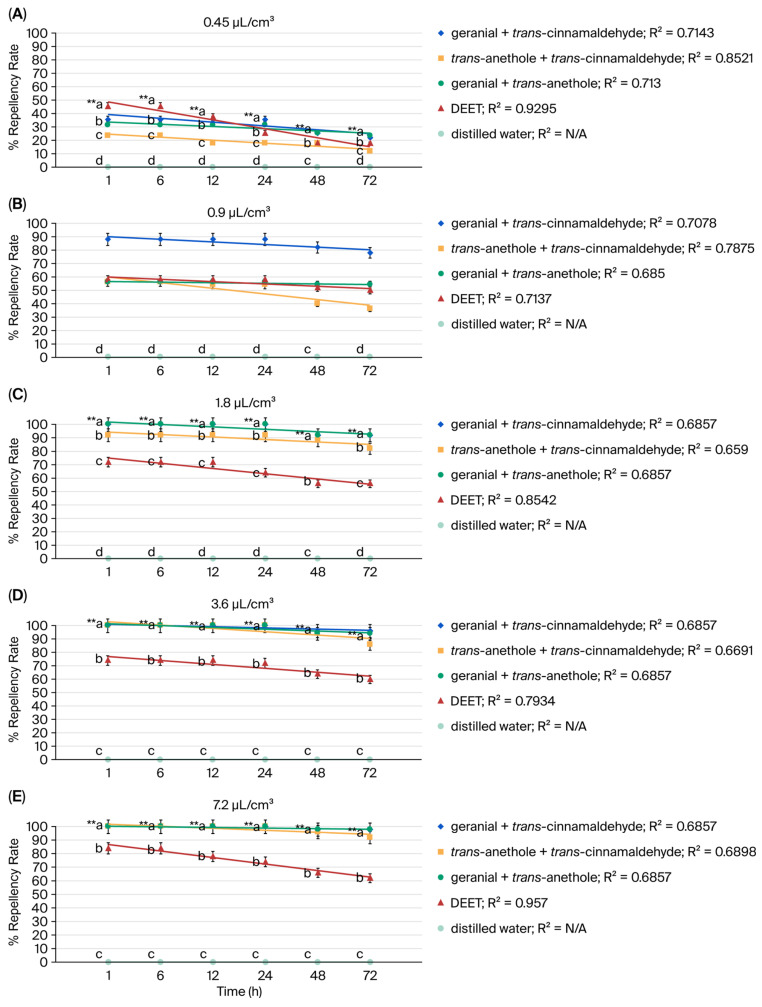
Repellency rate versus exposure time for terpenoid binary mixtures and DEET against the American cockroach (*P. americana*) at 0.45 µL/cm^3^ (**A**), 0.9 µL/cm^3^ (**B**), 1.8 µL/cm^3^ (**C**), 3.6 µL/cm^3^ (**D**), 7.2 µL/cm^3^ (**E**). Values that are accompanied by different letters (a–d) show significant differences between the treatments. ** for *p* < 0.01, the coefficient of determination value (R^2^).

Binary mixtures, geranial, *trans*-anethole, and *trans*-cinnamaldehyde exhibited strong repellent activity against adult American cockroaches (*P. americana*)—i.e., stronger than 12% (*w*/*w*) DEET. A repellency rate of 98% was demonstrated by all three terpenoid binary mixtures at 7.2 µL/cm^3^ at 72 h. Compared to the synthetic repellent DEET, which demonstrated only 62% efficacy at 72 h, the binary mixtures were much more repellent. For a single terpenoid, however, the maximum repellency was 56% at 72 h. The three terpenoid mixtures initially showed 100% repellency, declining by 2.0–8.0% at 72 h based on linear regression analysis. DEET, on the other hand, demonstrated an initial repellency rate of 84%, with the rate decreasing by roughly 26.0% at 72 h. Distilled water resulted in no repellency rate. A high degree of precision and dependability in the experimental results was indicated by the coefficient of determination (R^2^) value being close to 1 (Figure 3 and Figure 4).

The concentrations of treatments (RC_25_, RC_50_, and RC_90_) needed to repel 25%, 50%, and 90% of the adult American cockroaches (*P. americana*) after 72 h of exposure are shown in Table 2. It was discovered that all lone terpenes had an RC_25_ value between 2.0 and 3.1 µL/cm^3^, an RC_50_ value between 6.2 and 9.1 µL/cm^3^, and an RC_90_ value between 14.2 and 20.2 µL/cm^3^ after 72 h of exposure. On the other hand, terpenoid binary mixtures exhibited noticeably lower repellent concentrations, with RC_25_ values falling between 0.2 and 0.6 µL/cm^3^, RC_50_ values between 0.3 and 1.6 µL/cm^3^, and RC_90_ values between 1.1 and 4.6 µL/cm^3^. The binary mixtures outperformed DEET, which showed RC_25_, RC_50_, and RC_90_ values of 1.3, 3.0, and 6.5 µL/cm^3^, respectively. Also, the binary mixtures outperformed the lone terpenoid.

**Figure 5:**
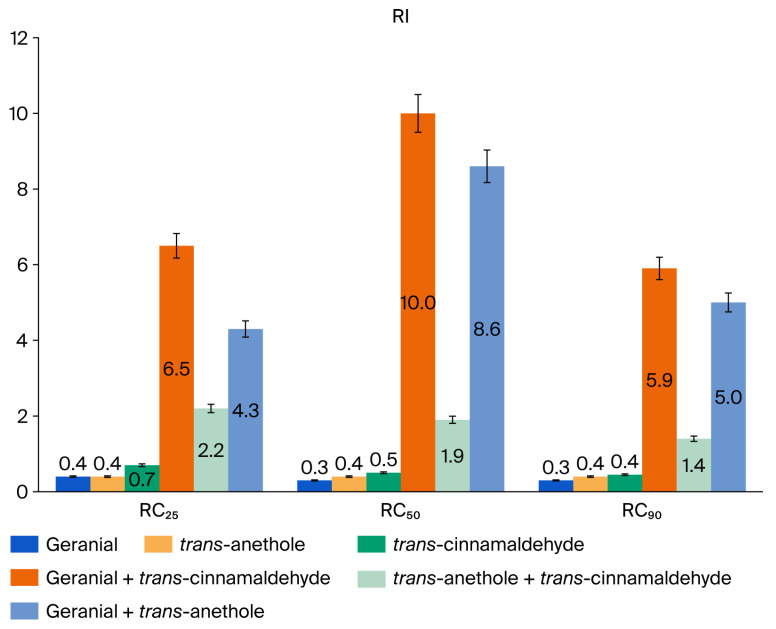
Comparative repellency index (RI) of lone terpenes, terpenoid binary mixtures, and DEET against the American cockroach (*P. americana*) at RC_25_, RC_50_, and RC_90_.

Terpenoid binary mixtures showed noticeably greater repellency against the American cockroach (*P. americana*) than lone terpenoids, as can be seen in Figure 5. The repellency index (RI) compares the efficacy of the binary mixture to that of DEET. The RIs for all the binary mixtures at RC_25_, RC_50_, and RC_90_ concentrations were 2.2–6.5, 1.9–10, and 1.4–5.9 times those of DEET, respectively. The RIs for the lone terpenoids, on the other hand, were significantly lower and only varied between 0.3 and 0.7 times the RI of DEET at all tested concentrations. According to these results, binary terpenoid mixtures must have acted synergistically, increasing their repellency beyond that of lone terpenoids.

**Figure 6:**
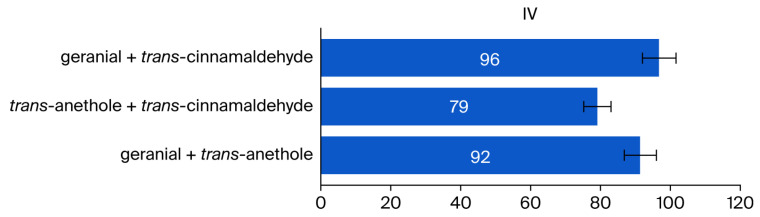
Increased repellency value (IV) of terpenoid binary mixtures against the American cockroach (*P. americana*) versus corresponding single terpenoids.

In comparison to the lone terpenes, the binary mixtures showed an increase in repellency against American cockroaches of roughly 79–96%, which indicates a strong synergy, as demonstrated in the results displayed in Figure 6.

The toxicities of the chemicals to guppy fish and earthworms, the non-target creatures used in this study, are shown in Table 3 and Table 4. A high degree of safety was demonstrated by the fact that neither the lone terpenes nor the terpenoid binary mixtures killed any non-target organisms during the 15-day exposure period. The chemical insect repellent DEET, on the other hand, caused 100% mortality, indicating that it is very harmful to non-target species. Distilled water resulted in no mortality.

## 4. Discussion

There is a high demand to develop natural pesticides and repellents for the management of pests and insect vectors, including the American cockroach, because of the global trends of rising human health issues caused by overuse of synthetic insecticides [8,41,42,43]. Furthermore, the extensive and frequently uncontrolled use of these chemicals has resulted in major problems like food source contamination and environmental, ecosystem, and agricultural pollution [44]. Given the serious dangers associated with both acute and chronic pesticide poisoning, exposure to these residues has become a major public health problem, especially in developing countries [44,45]. Synthetic repellents and insecticides also frequently impact non-target organisms, such as humans and beneficial animals, due to their broad range of action [46,47]. In this context, terpenes from essential oils have been identified as promising green alternatives to synthetic repellents for American cockroaches and other vector insects due to their widely reported repellent activity and their harmlessness to non-target predators, pollinators, and earthworms [19,23,31,48]. Our findings are significant in that terpenoid mixtures were found to exhibit significant potential as alternative American cockroach (*P. americana*) repellents.

These findings demonstrated that within 1 to 24 h of application, the terpenoid binary mixtures of geranial + *trans*-cinnamaldehyde, *trans*-anethole + *trans*-cinnamaldehyde, and geranial + *trans*-anethole all attained 100% repellency. At 72 h, there was a modest decrease in repellency. These mixtures’ RC_50_ values, ranging from 0.3 to 1.6, indicated high potency. In contrast, the lone terpenoids’ RC_50_ values ranged from 6.2 to 9.1, and their maximum repellency after 1 h was just 76%. Under the same conditions, DEET demonstrated a maximum repellency of 84% and an RC_50_ value of 3.0. These results show that the terpenoid binary mixtures provide noticeably higher repellent activity than the corresponding lone terpenes and are more effective than traditional synthetic repellents like DEET. The mixture of geranial and *trans*-cinnamaldehyde, as well as that of geranial and *trans*-anethole, demonstrated repellency rates that were up to 10 times higher than that of DEET. On the other hand, the lone terpenoid’s maximum repellency was only 0.7 times higher than that of DEET. These findings unequivocally show that the terpenoid mixtures outperformed the single-compound treatments in terms of their repellency, exhibiting synergistic effects.

In addition, several terpenoid mixtures (i.e., D-limonene + geranial, geranial + *trans*-cinnamaldehyde, and D-limonene + *trans*-cinnamaldehyde) presented highly synergistic repellent effects against the American cockroach and other medical insect pests, leading to repellents that are stronger than single-terpene formulations, with the additional benefits of reduced effective terpene concentration and cost [15,16,28,49,50,51]. In this study, all terpenoid binary mixtures, as opposed to lone terpenes, showed highly synergistic effects against American cockroach adults, with a higher repellency rate; lower RC_25,_ RC_50,_ and RC_90_ values; a higher repellency index (RI); and a higher increased repellent value (IV), with the terpenoid mixture of geranial + *trans*-cinnamaldehyde being especially promising. These findings are consistent with those of other studies that report strong synergistic effects. One study reported that a 1:1 EO mixture of star anise (*I. verum*, major composition is *trans*-anethole) and cinnamon (*C. verum*, major composition is *trans*-cinnamaldehyde) demonstrated strong repellency against American cockroach adults, with a 100% repellency rate at 1h after treatment [28]. The adult American cockroaches displayed knockdown and mortality from the mixture of eugenol + α-terpinol + cinnamic alcohol at a 1:1:1 ratio [51]. Similarly, the binary terpenoid mixture of geranial + *trans*-anethole at 0.5:0.5 and 1:1 ratios showed strong repellency, with highly synergistic effects, against housefly adults (*M. domestica*) [19]. Three binary mixtures of geranial + *trans*-cinnamaldehyde, eucalyptol + *trans*-anethole, and *trans*-anethole + geranial in a 1:1 ratio at 400–500 ppm showed highly synergistic larvicidal and pupicidal effects against the dengue mosquito, *Ae. aegypti* [17,18].

DEET (*N*,*N*-diethyl-meta-toluamide), a synthetic molecule, served as a reference compound in this experiment. The findings of this study show that DEET repelled American cockroaches at a far lesser rate than the terpenoid binary mixtures. Our repellency index (RI) showed that all terpenoid binary mixtures were 1.4 to 10 times more potent than DEET, similar to other reports [15,52,53,54]. One study reported that two binary EO mixtures, namely, star anise + cinnamon and star anise + lemon grass (*C. citratus*, the main component of which is geranial) at a 1:1 ratio, were more repellent against the American cockroach than DEET, with a repellency index of 1.1 to 1.6 [15]. The binary mixture of *trans*-anethole + geranial at a 1:1 ratio was twice as repellent against housefly adults than 12% DEET, showing 100% repellency at 6 h after treatment [19]. The natural mixtures simply outperformed DEET under these test conditions. 

Terpenoids interfere with insects’ odorant and gustatory receptors and respiratory systems [28,55] as well as the antennal olfactory receptors and other chemo-sensory receptors in the tarsal sensilla and palpi of mosquitoes, houseflies, and other insects [56,57]. In this way, the terpenoid mixture of geranial and *trans*-cinnamaldehyde exhibited a high repellency rate for the tested exposure periods. The mixture of geranial and *trans*-cinnamaldehyde not only demonstrated repellency but also an insecticidal effect. Other studies reported that they inhibited acetylcholinesterase (AChE) enzymes and the insect respiratory system, causing mortality [55,58,59,60,61].

Generally, terpenoids derived from several essential oils are regarded as environmentally friendly and harmless to non-target species such as honeybees, stingless bees, guppies, mollies, and earthworms [17,18,19,28,62]. In the present study, all individual terpene formulations and binary terpenoid mixtures were found to be non-toxic to guppies and earthworms after 15 days of exposure. Consistent with previous research, a mixture of geranial + *trans*-anethole was shown to be benign to the dwarf honeybee (*A. florea*), the stingless bee (*T. pagdeni*), the guppy (*P. reticulata*), and the molly (*P. latipinna*) [19]. Geranial and *trans*-anethole also exhibited low toxicity to honeybees (*A. mellifera*) and predatory bugs (*Podisus nigripinus*) [63,64]. Similarly, binary mixtures of geranial + *trans*-cinnamaldehyde (1.5:1.5), geranial + D-limonene (1.5:1.5), and *trans*-cinnamaldehyde + D-limonene (1.5:1.5) at high concentrations (10,000 ppm) were non-toxic to mollies and guppies [22]. The biosafety results against earthworms in this study were also consistent with those reported by Passara et al. [31], who demonstrated that a combination of anise (*P. anisum*) and fennel (*F. vulgare*) essential oils at a 1:1 ratio and concentrations of 100, 200, and 400 μL/kg was non-toxic to earthworms. Likewise, essential oils from *Satureja sahendica*, *S. khuzestanica*, *S. rechingeri* (Family Lamiaceae), and *Oliveria decumbens* (Family Apiaceae) at 200 mg/kg were shown to be safe for earthworms after 14 days of treatment [65].

In addition, geranial and *trans*-cinnamaldehyde are considered safe and valuable preventive agents for human health [32,66]. *Trans*-cinnamaldehyde exhibits multiple pharmacological properties, including antioxidant, anti-inflammatory, and antimicrobial activities and has been reported to support cardiovascular health and prevent the progression of cardiovascular disease [66]. Geranial likewise provides numerous benefits for humans and mammals, such as antimicrobial, antioxidant, anti-inflammatory, and potential anticancer effects. Moreover, it has been shown to promote metabolic health and improve meat quality [67,68].

In contrast, DEET at concentrations of 0.1, 0.5, and 1.0 mL/L, as well as 0.1, 0.5, and 1.0 mL/kg, exhibited pronounced toxicity to guppies and earthworms throughout the experiment. Consistent with previous reports, DEET has been shown to be harmful to aquatic environments and non-target species, including algae (*Pseudokirchneriella subcapitata*, *Chlorella protothecoides*), zooplankton and crustaceans (*Daphnia magna*, *Macrobrachium nipponense*), amphibians and fish (*Rhodeus sinensis*), and invertebrates (*Chironomus riparius*, *Limnodrilus hoffmeisteri*) [46,69]. More critically, DEET also poses risks to humans and mammals, with documented adverse effects such as seizures, vomiting, respiratory distress, skin irritation, and eye irritation [70,71].

The terpenoid binary mixture of geranial + *trans*-cinnamaldehyde demonstrated highly synergistic repellency activity. The mixture has a 96% increased repellency rate against adult American cockroaches and remains eco-friendly. Consequently, the strong repellency responses observed in the American cockroaches demonstrate that this formulation is a viable, sustainable, and safe substitute for synthetic repellents.

## 5. Conclusions

The findings of our investigation demonstrate that even at low concentrations, a terpenoid mixture of geranial + *trans*-cinnamaldehyde can effectively repel American cockroaches. This terpenoid mixture is also non-toxic to non-target species such as earthworms and guppies. Moreover, the mixture is both safer and more effective against American cockroaches than the synthetic insect repellent DEET. In terms of public health, it is clear from the results that this terpenoid binary mixture is well-positioned for future development into a commercially accessible and eco-friendly repellent for adult American cockroach populations and the infectious diseases they mechanically transmit.

## Figures and Tables

**Table 1:** Chemical composition of essential oils from *Cymbopogon citratus*, *Cinnamomum verum*, and *Illicium verum*.

No	Constituent ^a^	RI ^b^	KI ^c^	Percentage of Total Composition			ID ^d^
				*C. citratus*	*I. verum*	*C. verum*	
1	α-Thujene	931	930	–	0.22 ± 0.02	–	RI, Std
2	α-Pinene	949	949	3.40 ± 0.06	–	0.51 ± 0.04	RI, Std
3	Camphene	951	952	–	–	0.61 ± 0.04	RI, Std
4	Sabinene	967	969	–	–	–	RI, Std
5	β-pinene	979	979	–	–	–	RI, Std
6	β-Myrcene	990	991	–	–	0.32 ± 0.11	RI, Std
7	α-Phellandrene	1003	1003	–	–	0.42 ± 0.11	RI, Std
8	δ-3-Carene	1006	1006	–	–	–	RI, Std
9	Benzyl alcohol	1009	1009	–	–	12.5 ± 0.69	RI, Std
10	α-Terpinene	1012	1012	0.15 ± 0.02	–	0.21 ± 0.09	RI, Std
11	Limonene	1032	1032	–	1.85 ± 0.08	0.63 ± 0.09	RI, Std
12	1,8-Cineole	1033	1033	10.60 ± 0.03	0.71 ± 0.04	0.61 ± 0.06	RI, Std
13	(*E*)-β-Ocimene	1049	1050	–	–	–	RI, Std
14	γ-Terpinene	1051	1052	0.10 ± 0.01	–	–	RI, Std
15	Terpinolene	1089	1088	–	–	–	RI, Std
16	Linalool	1101	1101	0.80 ± 0.01	–	–	RI, Std
17	Terpinen-4-ol	1180	1179	–	–	–	RI, Std
18	α-Terpineol	1190	1191	–	0.21 ± 0.01	–	RI, Std
19	Neral	1216	1216	24.80 ± 4.62	–	–	RI
20	*trans*-Cinnamaldehyde	1221	1221	–	–	75.21 ± 2.73	RI, Std
21	Nerol	1232	1232	–	–	–	RI, Std
22	Geraniol	1235	1235	4.50 ± 0.00	–	–	RI, Std
23	Geranial	1246	1246	46.45 ± 2.26	–	–	RI, Std
24	*p*-Anisaldehyde	1247	1247	–	1.21 ± 0.08	–	RI, Std
25	Linalyl acetate	1261	1261	–	–	–	RI, Std
26	*trans*-Anethole	1283	1283	–	94.29 ± 2.04	–	RI, Std
27	Eugenol	1355	1355	–	0.51 ± 0.02	2.40 ± 0.86	RI, Std
28	Neryl acetate	1368	1368	–	–	–	RI, Std
29	α-Copaene	1378	1378	–	–	1.80 ± 0.41	RI
30	Geranyl acetate	1382	1381	3.30 ± 0.02	–	–	RI, Std
31	Cinnamyl acetate	1415	1414	–	–	2.30 ± 0.53	RI, Std
32	Cinnamic acid	1462	1462	–	–	0.50 ± 0.19	RI, Std
33	*trans*-Nerolidol	1566	1565	–	–	–	RI, Std
34	Caryophyllene oxide	1581	1581	2.50 ± 0.02	–	–	RI, Std
35	Cadalene	1658	1658	–	–	0.20 ± 0.05	RI
	Total identified (%)			96.60 ± 1.3	99.00 ± 0.95	98.22 ± 1.5	
	Color			Pale yellow	Pale yellow	Pale yellow	
	Yield (% *v*/*w*)			1.14	3.13 ± 0.08	1.05	

^a^ Constituents listed in order of elution in the HP-5MS column. ^b^ Retention index (*RI*) calculated through the retention time in relation to the series of C7–C30 n-alkanes. ^c^ Kovats retention index (*KI*) taken from https://pubchem.ncbi.nlm.nih.gov (accessed on 5 May 2025). ^d^ Identification method (*ID*): Substance matching (std) was performed with a readily available analytical standard (Sigma-Aldrich). RI value matching with those reported in NIST 17 is taken from https://webbook.nist.gov (accessed on 5 May 2025).

**Table 2:** Concentrations of single terpenes, terpenoid binary mixtures, and DEET that cause 25, 50 and 90% repellency in American cockroaches, *P. americana*, after 72 h of exposure.

Treatment	Repellent Concentration	Estimated Concentration (µL/cm^3^)	CI_95_ (µL/cm^3^)	Chi-Square	Slope ± SE
geranial	RC_25_	3.1	1.3–5.0	4.654	−2.532 ± 0.552
	RC_50_	9.1	6.5–12.9		
	RC_90_	20.2	13.7–25.1		
*trans*-anethole	RC_25_	2.9	1.7–4.1	6.687	−0.745 ± 0.495
	RC_50_	7.1	5.6–10.3		
	RC_90_	15.1	11.4–20.5		
*trans*-cinnamaldehyde	RC_25_	2.0	0.5–3.1	6.318	−1.694 ± 0.463
	RC_50_	6.2	4.9–8.8		
	RC_90_	14.2	10.9–22.4		
geranial + *trans*-cinnamaldehyde	RC_25_	0.2	0.05–0.3	138.573	−1.269 ± 1.156
	RC_50_	0.3	0.1–0.8		
	RC_90_	1.1	0.8–1.6		
*trans*-anethole + *trans*-cinnamaldehyde	RC_25_	0.6	0.2–0.8	56.402	−0.587 ± 0.767
	RC_50_	1.6	0.7–2.4		
	RC_90_	4.6	2.3–5.8		
geranial + *trans*-anethole	RC_25_	0.3	0.1–0.8	114.009	−1.529 ± 0.958
	RC_50_	0.7	0.2–1.2		
	RC_90_	1.3	0.8–1.9		
DEET	RC_25_	1.3	0.8–2.0	18.153	−1.668 ± 1.227
	RC_50_	3.0	1.3–4.8		
	RC_90_	6.5	3.4–8.6		

RC_25, 50, 90_ = concentration required for 25%, 50%, and 90% repellency. CI_95_ = 95% confidence intervals. The exposure concentration is considered significantly different when the 95% CI fails to overlap.

**Table 3:** Toxicity of different treatments to non-target earthworms (*E. eugeniae*) at 15 days after the beginning of the test.

Treatment	Concentration (mL/kg)	Mortality Rate (%) (Mean ± SD)			
		Day 1	Day 5	Day 10	Day 15
geranial	0.1	0 ^d^	0 ^d^	0 ^d^	0 ^d^
	0.5	0 ^d^	0 ^d^	0 ^d^	0 ^d^
	1.0	0 ^d^	0 ^d^	0 ^d^	0 ^d^
*trans*-anethole	0.1	0 ^d^	0 ^d^	0 ^d^	0 ^d^
	0.5	0 ^d^	0 ^d^	0 ^d^	0 ^d^
	1.0	0 ^d^	0 ^d^	0 ^d^	0 ^d^
*trans*-cinnamaldehyde	0.1	0 ^d^	0 ^d^	0 ^d^	0 ^d^
	0.5	0 ^d^	0 ^d^	0 ^d^	0 ^d^
	1.0	0 ^d^	0 ^d^	0 ^d^	0 ^d^
geranial + *trans*-cinnamaldehyde	0.1	0 ^d^	0 ^d^	0 ^d^	0 ^d^
	0.5	0 ^d^	0 ^d^	0 ^d^	0 ^d^
	1.0	0 ^d^	0 ^d^	0 ^d^	0 ^d^
*trans*-anethole + *trans*-cinnamaldehyde	0.1	0 ^d^	0 ^d^	0 ^d^	0 ^d^
	0.5	0 ^d^	0 ^d^	0 ^d^	0 ^d^
	1.0	0 ^d^	0 ^d^	0 ^d^	0 ^d^
geranial + *trans*-anethole	0.1	0 ^d^	0 ^d^	0 ^d^	0 ^d^
	0.5	0 ^d^	0 ^d^	0 ^d^	0 ^d^
	1.0	0 ^d^	0 ^d^	0 ^d^	0 ^d^
distilled water	0.1	0 ^d^	0 ^d^	0 ^d^	0 ^d^
	0.5	0 ^d^	0 ^d^	0 ^d^	0 ^d^
	1.0	0 ^d^	0 ^d^	0 ^d^	0 ^d^
DEET	0.1	15.0 ± 2.7 ^c^	21.5 ± 3.7 ^c^	21.5 ± 3.7 ^c^	25.5 ± 2.9 ^c^
	0.5	56.0 ± 4.7 ^b^	56.0 ± 4.7 ^b^	60.0 ± 3.8 ^b^	60.0 ± 3.8 ^b^
	1.0	96.0 ± 4.2 ^a^	100.0 ± 0.0 ^a^	100.0 ± 0.0 ^a^	100.0 ± 0.0 ^a^
	ANOVA*_F_*_0.01_, D*f_total_*	**, 119	**, 119	**, 119	**, 119
	*p*-value	*p* < 0.01	*p* < 0.01	*p* < 0.01	*p* < 0.01

Note: Mortality rates within a column with the same letters (a–d) do not differ significantly between the treatments (Tukey’s post hoc test *p* < 0.01). ** for *p* < 0.01.

**Table 4:** Toxicity of different treatments to non-target guppies (*P. reticulata*) at 15 days after the beginning of the test.

Treatment	Concentration (mL/L)	Mortality Rate (%) (Mean ± SD)			
		Day 1	Day 5	Day 10	Day 15
geranial	0.1	0 ^d^	0 ^d^	0 ^d^	0 ^d^
	0.5	0 ^d^	0 ^d^	0 ^d^	0 ^d^
	1.0	0 ^d^	0 ^d^	0 ^d^	0 ^d^
*trans*-anethole	0.1	0 ^d^	0 ^d^	0 ^d^	0 ^d^
	0.5	0 ^d^	0 ^d^	0 ^d^	0 ^d^
	1.0	0 ^d^	0 ^d^	0 ^d^	0 ^d^
*trans*-cinnamaldehyde	0.1	0 ^d^	0 ^d^	0 ^d^	0 ^d^
	0.5	0 ^d^	0 ^d^	0 ^d^	0 ^d^
	1.0	0 ^d^	0 ^d^	0 ^d^	0 ^d^
geranial + *trans*-cinnamaldehyde	0.1	0 ^d^	0 ^d^	0 ^d^	0 ^d^
	0.5	0 ^d^	0 ^d^	0 ^d^	0 ^d^
	1.0	0 ^d^	0 ^d^	0 ^d^	0 ^d^
*trans*-anethole + *trans*-cinnamaldehyde	0.1	0 ^d^	0 ^d^	0 ^d^	0 ^d^
	0.5	0 ^d^	0 ^d^	0 ^d^	0 ^d^
	1.0	0 ^d^	0 ^d^	0 ^d^	0 ^d^
geranial + *trans*-anethole	0.1	0 ^d^	0 ^d^	0 ^d^	0 ^d^
	0.5	0 ^d^	0 ^d^	0 ^d^	0 ^d^
	1.0	0 ^d^	0 ^d^	0 ^d^	0 ^d^
distilled water	0.1	0 ^d^	0 ^d^	0 ^d^	0 ^d^
	0.5	0 ^d^	0 ^d^	0 ^d^	0 ^d^
	1.0	0 ^d^	0 ^d^	0 ^d^	0 ^d^
DEET	0.1	25.0 ± 3.8 ^c^	30.0 ± 4.7 ^c^	30.0 ± 4.7 ^c^	35.0 ± 4.9 ^c^
	0.5	60.0 ± 3.7 ^b^	60.0 ± 3.7 ^b^	65.0 ± 4.8 ^b^	65.0 ± 4.8 ^b^
	1.0	98.0 ± 3.3 ^a^	100.0 ± 0.0 ^a^	100.0 ± 0.0 ^a^	100.0 ± 0.0 ^a^
	ANOVA*_F_*_0.01_, D*f_total_*	**, 119	**, 119	**, 119	**, 119
	*p*-value	*p* < 0.01	*p* < 0.01	*p* < 0.01	*p* < 0.01

Note: Mortality rates within a column with the same letters (a–d) do not differ significantly between the treatments (Tukey’s post hoc test *p* < 0.01). ** for *p* < 0.01.

## Data Availability

The original contributions presented in this study are included in the article. Further inquiries can be directed to the corresponding author.
